# Association of Dietary Patterns with Excess Weight and Body Adiposity in Brazilian Children: The Pase-Brasil Study

**DOI:** 10.5935/abc.20190113

**Published:** 2019-07

**Authors:** Naruna Pereira Rocha, Luana Cupertino Milagres, Mariana De Santis Filgueiras, Lara Gomes Suhett, Mariane Alves Silva, Fernanda Martins de Albuquerque, Andréia Queiroz Ribeiro, Sarah Aparecida Vieira, Juliana Farias de Novaes

**Affiliations:** Departamento de Nutrição e Saúde - Universidade Federal de Viçosa, Viçosa, MG - Brazil

**Keywords:** Child, Obesity, Adiposity, Hyperphagia, Feeding Behavior, Factor Analysis, Statistical, Epidemiology

## Abstract

**Background:**

Obesity is a multifactorial disease and a serious public health problem. Some of the associated factors are modifiable and, among them, the diet is highlighted.

**Objective:**

To evaluate the association of dietary patterns of schoolchildren with obesity and body adiposity.

**Methods:**

A cross-sectional study was carried out with 378 children aged 8 and 9 years, enrolled in urban schools in the city of Viçosa, Minas Gerais, Brazil. A semi-structured questionnaire was applied to the children and their caregivers on sociodemographic characteristics and life habits. Three 24-hour food recalls were used to identify dietary patterns; the Principal Component Analysis was employed. Weight and height were measured for the calculation of the body mass index (BMI) of the children and their mothers, waist circumference and neck circumference. Body composition was also evaluated through dual-energy X-ray absorptiometry (DXA). For all performed tests, the level of significance was set at 5%.

**Results:**

Five dietary patterns (DP) were identified: “unhealthy”, “snacks”, “traditional”, “industrialized” and “healthy”. There was an association between excess weight (prevalence ratio [PR]: 1.38, 95% confidence interval [95%CI]: 1.02 to 1.87) and body fat (PR: 1.32, 95%CI : 1.07 to 1.64) with industrialized DP. There was an association between excess body fat (PR: 1.31, 95%CI: 1.01 to 1.74) and lower adherence to traditional DP. The other patterns were not associated with obesity and body adiposity.

**Conclusion:**

Children with excess weight and body adiposity showed greater adherence to the industrialized DP and lower adherence to the traditional DP. We suggest that early assessments of dietary habits should be undertaken for monitoring and modifying these habits when necessary.

## Introduction

Childhood obesity is considered a serious public health problem.^1^ According to the World Health Organization (WHO, 2016),^2^ approximately 41 million children worldwide under five years of age have excess weight or obesity. In Brazil, the prevalence of obesity is increasing, and data related to childhood excess weight show that it is increasing in children from five to nine years of age more rapidly than in other age groups.^3^

Obesity can be considered a multifactorial disease due to genetic predisposition, sedentary lifestyle, availability of food inside and outside the home environment, inadequate and structural food patterns, such as food production and distribution systems, all of which play important roles in the etiology of this alteration.^4,5^

Among the several risk factors for obesity, the diet constitutes a modifiable factor and has been related to the onset of chronic diseases and cardiometabolic alterations.^6,7^ Several studies have shown that energetically dense, fiber-poor and refined carbohydrate-rich diets are associated with obesity in adults.^8,9^ In children, most of the results are based on the analysis of isolated foods or nutrients, and studies on dietary patterns are scarce.^10,11^

The analysis of dietary patterns allows the evaluation of the diet from a global perspective, favoring the implementation of strategies to promote healthy eating habits and prevention of nutrient-related diseases and conditions.^12^ As childhood is a period of formation of eating habits,^13^ initiatives that allow identifying inappropriate dietary practices and associating them with excess body weight and adiposity could help to prevent chronic diseases, as well as to reduce short and long-term health damage by encouraging and adopting healthy habits.^14^

Considering the above, the aim of this study was to evaluate the association of eating patterns of schoolchildren with obesity and body adiposity. Our hypothesis is that unhealthy eating patterns are associated with excess weight and body adiposity in Brazilian children.

## Methods

### Study population and design

This is a cross-sectional study, with a representative sample of 378 children from public and private schools in the urban area of the municipality of Viçosa, state of Minas Gerais, Brazil. The participants of this study came from the Schoolchildren Health Assessment Survey (PASE, *Pesquisa de Avaliação da Saúde do Escolar*). The PASE aimed to investigate the cardiovascular health of children in the city of Viçosa, MG.

The municipality of Viçosa is located in Zona da Mata Mineira, 227 km from the capital city, Belo Horizonte. Viçosa has a land area of 299 km^2^ and 72,244 inhabitants, with 67.3% residing in the urban area.^15^

In 2015, the municipality had 17 public schools and 7 private ones, which were attended by 8- and 9-year-old children in the urban area, totaling a population of 1,464 children enrolled in these schools. The sample was calculated using the OpenEpi statistical program (Version 3.01), considering a prevalence of 11.8% for obesity in the age group,^16^ a tolerable error of 5%, plus 10% of losses and 10% of confounding factors, totalizing a sample size of 392 children. The final sample included 378 children, with a sample loss of 3.6%. The losses were due to non-compliance with all stages of the study.

The sampling process was carried out in two stages. First, stratified casual sampling was performed, in which the number of children to be sampled from each school was proportional to the total number of students in each school. Subsequently, the lots were drawn by using a random number table, to complete the required number of students from the 24 public and private schools in the urban area that were attended by the assessed age group.

The non-inclusion criteria for this study were the lack of contact with the parents or guardians after three attempts to contact them, children with some clinical or nutritional alterations that could interfere with food consumption, nutritional status and body composition, as well as children with physical, cognitive or multiple disabilities.

This study was carried out in accordance with the guidelines established in the Declaration of Helsinki and was initiated only after approval by the Ethical Committee for Research in Human Beings of Universidade Federal de Viçosa (UFV) (Opinion n. 663.171/2014). The study was also approved by the Municipal Education Secretariat, Regional Superintendence of Education and the school principals. All parents and children were informed about the purpose of the study, and all the children’s parents/guardians signed the Free and Informed Consent form.

### Food consumption

Food consumption assessment was performed by applying three 24-hour food recalls (R24h) on non-consecutive days, including one weekend day and a 15-day mean interval between them, based on information provided by the mother/guardian and by the child. For the children who consumed part of the food in the school environment, the researchers obtained the information from the schools, such as recipes and portions of the foods offered, in addition to confirming with the children what had been consumed. For those children who used to bring their food from home, the parents/guardians were asked about food and beverages offered and their amounts. The three food recalls were applied by trained nutritionists.

The food consumption data obtained through the three R24h were tabulated and processed in Dietpro® 5i software, version 5.8.^17^

### Anthropometric data

All anthropometric measurements were performed by a trained nutritionist, selected after calibration of the team members. Weight was measured using a digital electronic scale, with a capacity of 150 kg and sensitivity of 50 g (Tanita®, model BC 553, Arlington Heights, IL, USA). Height was measured using a vertical stadiometer, divided in centimeters and subdivided in millimeters (Alturaexata®, Belo Horizonte, MG, Brazil).

The nutritional status was assessed by body mass index (BMI), with BMI cutoff points by age calculated in z-scores according to the World Health Organization (WHO) parameters (2007)^18^ as thinness, normal weight, overweight and obesity. Excess weight was considered for the overweight and obesity categories.

The waist circumference was obtained by measuring the midpoint between the iliac crest and the last rib using an inelastic measuring tape, divided in centimeters and subdivided in millimeters. Abdominal obesity was considered when the waist circumference was equal to the 90^th^ percentile of the sample itself, following the guidelines of the International Diabetes Federation (2007).^19^

The waist-to-height ratio (WHtR) was obtained by the waist circumference measurement divided by height. The cutoff point ≥ 0.5 was used as the risk for the development of cardiovascular diseases.^20^

The neck circumference (NC) was measured at the level of the thyroid cartilage using an inelastic measuring tape, divided in centimeters and subdivided in millimeters. The cutoff points proposed by Nafiu et al. (2010)^21^ for the detection of excess body fat in children were used for NC classification.

The children’ body composition was assessed through DXA by a specialized technician, obtaining the fat mass measurement. The children were evaluated in the morning, in fasting condition, in the supine position. Body fat was classified using the cutoff points proposed by Lohman (1992),^22^ and the cutoff points for overweight risk and overweight were considered as excess body fat.

### Adjustment variables

Potential adjustment variables were selected according to the previous literature.^23-25^ The collection of these variables was performed by nutritionists, using a questionnaire created by the researchers themselves. The questionnaire was previously tested in a pilot study, and the sample consisted of children aged 8 and 9 years.

The assessed sociodemographic variables were gender and self-reported ethnicity of the child, maternal schooling, family and *per capita* income, and the type of school in which the child was enrolled (public or private).

The behavioral variables were omission of breakfast and sedentary behavior. All questions were answered by parents or guardians. Breakfast consumption was assessed by the first food intake that the child consumed and/or drank within the first 2 hours after waking up.^24^

Sedentary behavior was assessed as time spent by the child in activities that did not increase energy expenditure, such as watching television or engaging in other forms of screen-based entertainment. The used cutoff point was screen time ≥2 hours/day, according to the American Academy of Pediatrics (2013).^25^

Maternal weight and height were measured using an electronic digital scale, with a capacity of 150 kg and sensitivity of 50 g (Tanita®, model BC 553, Arlington Heights, IL, USA) and a vertical stadiometer, divided in centimeters and subdivided in millimeters (Alturaexata®, Belo Horizonte, MG, Brazil), respectively. Using these data, it was possible to calculate the BMI and to classify it according to the WHO parameters (1998).^26^

### Statistical analysis

Descriptive statistics were used to characterize the sample according to sociodemographic, behavioral characteristics, nutritional status and body composition. At this phase, each variable was assessed through the distribution of absolute and relative frequencies.

The normality of the variables was evaluated by the Shapiro-Wilk test, in addition to the evaluation of graphical methods (histogram), kurtosis and asymmetry verification to classify variables regarding normality.

Aiming to identify the food pattern, the foods covered by the R24h were measured in grams/day (g/d) or milliliters/day (mL/d) and collected as isolated foods or food groups by nutritional similarity and their contribution to the hypothesis of diet-disease associations. Moreover, foods consumed by less than 10% of the assessed population were excluded or grouped.^27^ To identify the patterns, all foods in milliliters/day were transformed into grams/day according to the density table of the Food and Agriculture Organization (FAO, 2012).^28^

Pattern identification was carried out using an *a posteriori* methodology, through the Principal Component Analysis (PCA). Before starting the analysis, the sample size was carefully evaluated in relation to the food groups formed in the PCA analyses.^29^

For the PCA analysis, the results of the Kaiser-Meyer-Olkin test (KMO = 0.58) and the Bartlett sphericity test (p < 0.001) were estimated. They evaluate whether the data can be used in the PCA.^29^ The varimax rotation was performed to facilitate the interpretation of the obtained results, in which factorial loads ≥0.25 (positive or negative) were retained.^24^ The number of extracted factors was defined according to the *eigenvalue* criterion >1 followed by the scree plot graph of variance by the number of components, in which the points on the maximum slope indicated the number of components to be retained. The nomenclature of the found patterns was attributed according to the characteristics of the foods/ formed groups and extracted by PCA.

Dietary patterns were presented as explanatory variables, and the schoolchildren’s dietary patterns scores were categorized according to the 75^th^ percentile of the sample.

The Mann-Whitney test was performed to compare the medians of the anthropometric variables and body composition according to the classification of the dietary patterns.

The crude analysis was performed using Poisson regression models with robust variance, having anthropometry and body composition as dependent variables. The variables considered important in the assessed association were used for the model adjustment, such as gender, maternal BMI, total energy consumption (kcal) and breakfast consumption.

The prevalence ratio (PR) with 95% confidence interval (95% CI) was used as a measure of association. For all performed tests, the level of significance was set at 5%. Statistical analyses were performed using the Stata program version 13.0.

## Results

It was observed that more than half of the children had a sedentary behavior (74.9%) and mothers with excess weight (56.9%). Breakfast omission was observed in almost 20.0% of the sample (Table 1).

**Table 1:** Sample characterization according to socioeconomic, behavioral variables and maternal nutritional status of the children. Viçosa, MG, Brazil, 2015

Variable	N	%
**Age**		
8 years	183	48.4
9 years	195	51.6
**Gender**		
Female	197	52.1
Male	181	47.9
**Child Ethnicity /skin color**		
White	119	31.5
Non-white	259	68.5
**Type of School**		
Public	268	70.9
Private	110	29.1
**Maternal schooling**		
> 9 years	234	62.2
= 9 years	142	37.8
***per capita*** ** income tertiles**		
= 1500.0	133	35.2
> 1599.0 to 2340.98	117	31.0
> 2340.98	128	33.8
**Screen time**		
< 2 hours/day	95	25.1
= 2 hours/day	283	74.9
**Maternal excess weight**		
No	127	43.1
Yes	168	56.9
**Breakfast omission**		
No	303	80.2
Yes	75	19.8

The PCA analysis identified five dietary patterns (DP): (i) unhealthy DP, consisting of foods/groups of simple sugars and chocolate, fat-rich snacks and whole dairy foods; (ii) snacks DP, consisting of bakery products/food groups and infusions; (iii) traditional DP, consisting of rice, beans, flours, tubers and cereals; (iv) industrialized DP, consisting mainly of ultra-processed products; (v) healthy DP, consisting of foods rich in complex carbohydrates and high biological value proteins (Table 2).

**Table 2:** Distribution of factorial loads for the five identified food patterns. Viçosa, MG, Brazil, 2015

Foods	Dietary Patterns				
	**Unhealthy**	**Snacks**	**Traditional**	**Industrialized**	**Healthy**
Breads, biscuits and cakes without frosting		0.797			
Milk and dairy products	0.663				
Rice	-0.432		0.592		
Beans			0.61		
Sugar and chocolate milk	0.765				
Infusions	-0.555	0.361			
Butter and margarine		0.632			
Fruits and natural fruit juice				-0.519	0.295
Pasta			-0.258	0.276	
Flours, tubers and cereals			0.629		
Meat and eggs					0.507
Fat-rich snacks and sauces	0.428				
Vegetables				-0.307	0.679
Green vegetables					0.693
Sweets, candy		-0.256		0.448	
Artificial beverages				0.763	
Number of Items	5	4	4	5	4
Eigenvalues	2.30	1.72	1.50	1.25	1.10
% Explained variance	11.53	9.99	9.97	9.02	8.79
Total explained variance	49.33				
Kaiser-Meyer-Olkin (KMO) = 0,58 por:					
Kaiser-Meyer-Olkin (KMO) = 0.58					

Higher median BMI values (p = 0.001), body fat percentage (p = 0.002), waist circumference (p = 0.004), waist-to-height ratio (p = 0,030) and neck circumference (p = 0.001) were observed in children with higher consumption of the industrialized DP (Table 3).

**Table 3:** Median (IQ) of the anthropometric variables and body composition, according to the consumption percentiles of children’s dietary patterns. Viçosa, MG, Brazil, 2015

	Unhealthy DP		Snacks DP		Traditional DP		Industrialized DP		Healthy DP	
	**<p75**	**≥p75**	**<p75**	**≥p75**	**<p75**	**≥p75**	**<p75**	**≥p75**	**<p75**	**≥p75**
BMI	16.5	16.53	16.49	16.75	16.65	16.25	16.22	17.94	16.49	16.78
	(15.0-19.3)	(15.09-19.57)	(15.0-19.4)	(15.1-19.1)	(14.9-19.3)	(15.3-19.8)	(14.9-18.7)	(15.8-21.0)*	(14.9-19.6)	(15.3-19.1)
%BF	17.6	18.2	19.1	16.7	18.7	17	16.7	22.5	17.7	19.7
	(10.8-29.3)	(11.6-27.0)	(11.1-29.3)	(10.9-27.4)	(10.6-29.0)	(11.6-30.0)	(10.6-26.1)	(12.0-32.2)*	(10.6-29.2)	(12.5-29.0)
WC	59.6	60.0	59.0	60.5	59.0	59.0	58.0	62.0	58.8	59.7
	(54.8-68.8)	(55.6-68.2)	(55.0-68.5)	(55.0-68.8)	(54.4-68.1)	(55.5-69.1)	(54.5-66.0)	(56.5-72.0)*	(54.7-68.1)	(55.6-68.8)
WHtR	0.19	0.19	0.19	0.18	0.19	0.19	0.18	0.20	0.19	0.19
	(0.1-0.2)	(0.1-0.2)	(0.1-0.2)	(0.16-0.21)	(0.2-0.2)	(0.15-0.21)	(0.1-0.2)	(0.1-0.3)*	(0.1-0.2)	(0.1-0.2)
NC	26.9	27.3	26.9	27.2	26.9	27.4	26.8	27.7	27	27.3
	(25.9-28.3)	(26.3-28.6)	(26.0-28.3)	(26.0-28.8)	(25.9-28.3)	(26.0-28.5)	(25.7-28.1)	(26.5-29.0)*	(25.9-28.5)	(26.0-28.5)

*DP: Dietary Pattern; IQ: interquartile range for the 25th and 75th percentiles; BMI: body mass index; %BF: percentage of body fat; WC: waist circumference; WHtR: waist-to-height ratio; NC: neck circumference. Mann-Whitney test.*

* *Statistical significance (p <0.05)*

In the crude analysis of the regression model, a higher prevalence of increased neck circumference was found in children with higher consumption of snacks DP (PR: 1.79; 95% CI: 1.13 to 2.85). Children with excess weight (PR: 1.58; 95% CI: 1.18 to 2.10) and body fat (PR: 1.50; 95% CI: 1.23 to 1.82) showed higher adherence to the industrialized DP (Table 4).

**Table 4:** Unadjusted association between adiposity measures and dietary patterns in children. Viçosa, MG, Brazil, 2015

Dietary Patterns	Excess weight		Increased WC		Increased WHtR		Increased NC		% Increased body fat	
	**PR**	**95% CI**	**PR**	**95% CI**	**PR**	**95% CI**	**PR**	**95% CI**	**PR**	**95% CI**
Unhealthy DP	0.90	(0.64-1.28)	0.79	(0.37-1.67)	0.95	(0.57-1.59)	1.26	(0.76-2.07)	1.07	(0.86-1.35)
Snacks	1.12	(0.81-1.54)	1.21	(0.62-2.35)	1.11	(0.68-1.82)	1.79	(1.13-2.85)*	0.91	(0.71-1.16)
Traditional	0.96	(0.69-1.34)	0.82	(0.42-1.59)	0.96	(0.58-1.59)	1.03	(0.60-1.76)	1.20	(0.93-1.55)
Industrialized	1.58	(1.18-2.10)*	1.73	(0.93-3.22)	1.59	(1.01-2.49)	1.44	(0.89-2.34)	1.50	(1.23-1.82)*
Healthy	0.92	(0.66-1.27)	0.72	(0.38-1.38)	1.04	(0.62-1.75)	0.94	(0.56-1.59)	0.92	(0.73-1.16)

PR: prevalence ratio; 95% CI: confidence interval; WC: waist circumference; WHtR: waist-to-height ratio. Food standard assessed through the 75th percentile. For the traditional and healthy standards, the Percentile ≥ 75 was adopted as a protection factor, for the other standards the percentile < 75 was adopted as a reference.

* Statistical significance (p <0.05). Poisson regression with robust variance (bivariate).

**Figure 1:**
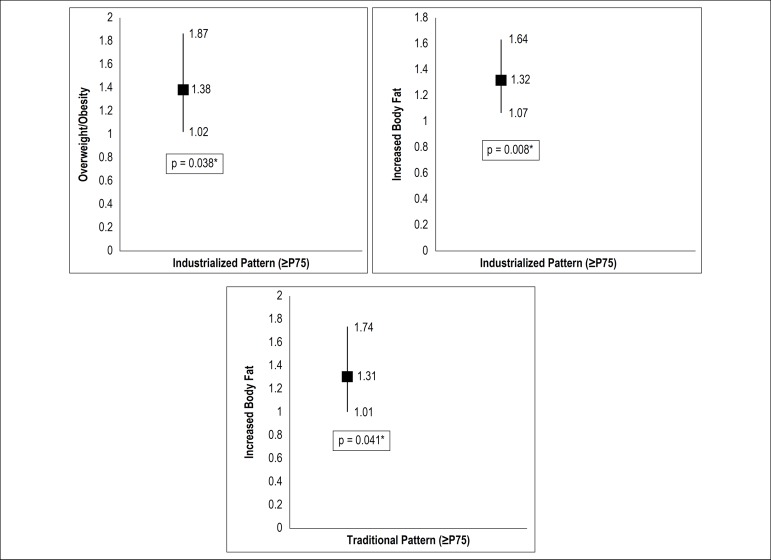
Association between dietary patterns and adiposity in children. Viçosa, MG, Brazil, 2015.

After the adjusted regression analysis, it was observed that children with excess weight (PR: 1.38, 95%CI: 1.02 to 1.87) and body fat (PR: 1.32, 95%CI: 1.07 to 1.64) showed greater adherence to the industrialized DP. Additionally, children with excess body fat (PR: 1.31; 95%CI: 1.01 to 1.74) showed lower adherence to the traditional DP (Figure 1).

## Discussion

In the present study, five dietary patterns were identified: unhealthy, snacks, traditional, industrialized and healthy. Children with excess weight and body adiposity showed greater adherence to the industrialized DP. Moreover, children with lower adherence to the traditional DP had higher adiposity prevalence.

The comparison of dietary patterns from different studies is difficult to make due to cultural, geographical and methodological differences.^5^ However, despite the complexity, the dietary patterns identified in this study are similar to those found in other national and international studies.^30,31^ Among Brazilian children aged 8 and 9 years, Villa et al. (2015)^31^ identified five food patterns: traditional pattern, consisting of rice, beans, roots and tubers and beef; DP of sweetened beverages and snacks, characterized by ultra-processed foods with high fat content and refined sugars; monotonous pattern, consisting of whole milk and chocolate; healthy pattern, characterized by the consumption of fibers and white meats; and the ovo-lacto pattern, characterized by the consumption of eggs, cheeses and sweetened milk-based beverages. Ambrosini et al. (2012)^10^ identified an energetically dense, high-fat, low-fiber DP in children and adolescents from 7 to 13 years of age. Durão et al. (2017)^30^ identified three patterns at 4 years of age, named energetically dense, snacks and healthy. Overall, industrialized DPs, rich in fats and refined carbohydrates, predominate in this population.

It is worth noting that the unhealthy pattern includes the participation of whole dairy products, foods that are recommended in childhood to guarantee the adequate supply of calcium and high biological value protein, essential for adequate growth in childhood.^32^ However, in the assessed population, milk consumption is attained with the addition of chocolate-flavored powder and simple sugars. This habit is common in childhood, but may lead to the consumption of a hypercaloric diet, predisposing to the risk of obesity and cardiometabolic alterations.^32,33^

The industrialized DP identified in this study consists of processed and ultra-processed foods, rich in simple sugars and fats, nutrients that favor lipogenesis, excess weight and an increase in metabolic complications in childhood.^10,34^ In this study, the prevalence of excess of body weight and adiposity were higher in the children with higher consumption of the industrialized DP. Studies have reported that low fruit and vegetable intake, associated with increased intake of fats and processed foods may increase the risk of obesity.^6,30^ A possible explanation for this association is that the usual consumption of a high-fat diet tends to impair appetite control, leading to hyperphagia due to the greater palatability of fatty foods, resulting in higher energy consumption.^35^ The higher consumption of fat, simple sugars and sweetened beverages by individuals with excess weight may also be explained by their lower effect on satiety when compared to other macronutrients.^35^

Children with excess body fat showed lower adherence to the traditional DP. The traditional pattern identified in this study is characterized by the consumption of rice and beans, as well as other carbohydrates. Beans are a legume source of soluble and insoluble proteins, minerals, vitamins, and soluble and insoluble fibers, which, when routinely consumed, may be associated with a reduced risk of cardiovascular diseases.^36^ Kupek et al. (2016),^37^ when evaluating dietary patterns in schoolchildren aged 7 to 10 years, concluded that children who consumed rice and beans had a lower risk of obesity.

Some studies evaluating the association between dietary patterns and body composition in children found similar results to ours. Durão et al. (2017)^30^ observed that girls with the highest adherence to the high-energy density diet, consisting mainly of sweets, soft drinks, pastries and processed meats, had higher values of BMI, WHtR and body fat. Zhang et al. (2015),^38^ assessing Chinese children and adolescents, found that the modern dietary pattern of northern China was associated with an increased risk of obesity. Shang et al. (2012)^7^ observed that obesity was more prevalent in children who adopted the western dietary pattern, when compared to those who consumed the traditional healthy pattern.

Our findings highlight the importance of assessing dietary patterns in the population, especially in children, who may, from an early age, have inadequate eating habits that promote excess weight.^11,23^ The presence of obesity in children may increase the risk for developing cardiovascular diseases and predict health risks later in life.^7^

As limitations of our study, we can highlight the evaluation of dietary patterns through the 24-hour food recall, which may underestimate the actual consumption of children due to memory bias and/or lack of cooperation of the interviewee. However, we emphasize that all R24h were applied by properly trained nutritionists. Moreover, the child was present with the person responsible for answering the dietary survey, since children under 12 years of age might not answer accurately regarding food intake information.

Some strong points of this study should be highlighted. This is one of the few studies in developing countries that investigated the association between dietary patterns and adiposity in childhood. As the consumption of industrialized foods contributes to excess weight and body fat, this is an important step in assessing dietary patterns, since diet is a modifiable risk factor for cardiovascular disease. These findings are consistent with other studies, suggesting that the consumption of industrialized foods is increasing, and these are already associated with cardiometabolic alterations in the early stages of life, such as in childhood.

## Conclusion

It was concluded that the prevalence of excess weight and body adiposity were higher in children with greater adherence to the industrialized DP. The lower consumption of the traditional DP was associated with excess body adiposity. Our study suggests that early assessments of eating habits should be undertaken for monitoring and modifying these habits, when necessary. Parents and health professionals need to be aware of the high consumption of processed and ultra-processed products by children. Food and nutritional educational actions become of the utmost importance in schools as a way to reinforce the healthy diet of children and their parents.
